# Molecular profile of *KRAS* G12C-mutant colorectal and non-small-cell lung cancer

**DOI:** 10.1186/s12885-021-07884-8

**Published:** 2021-02-25

**Authors:** Luiz Henrique Araujo, Bianca Mendes Souza, Laura Rabelo Leite, Sabrina A. F. Parma, Natália P. Lopes, Frederico S. V. Malta, Maíra C. M. Freire

**Affiliations:** 1 Progenética, Grupo Pardini, Vespaziano, Brazil; 2 Research & Development Sector, Grupo Pardini, Vespaziano, Brazil; 3 Instituto COI de Educação e Pesquisa, Rio de Janeiro, Brazil; 4 Instituto Nacional de Câncer, Rio de Janeiro, Brazil

**Keywords:** Lung neoplasms, Colorectal neoplasms, KRAS, Molecular targeted therapy, Molecular diagnostics

## Abstract

**Background:**

*KRAS* is the most frequently mutated oncogene in cancer, however efforts to develop targeted therapies have been largely unsuccessful. Recently, two small-molecule inhibitors, AMG 510 and MRTX849, have shown promising activity in *KRAS* G12C-mutant solid tumors. The current study aims to assess the molecular profile of *KRAS* G12C in colorectal (CRC) and non-small-cell lung cancer (NSCLC) tested in a clinical certified laboratory.

**Methods:**

CRC and NSCLC samples submitted for *KRAS* testing between 2017 and 2019 were reviewed. CRC samples were tested for *KRAS* and *NRAS* by pyrosequencing, while NSCLC samples were submitted to next generation sequencing of *KRAS*, *NRAS*, *EGFR*, and *BRAF*.

**Results:**

The dataset comprised 4897 CRC and 4686 NSCLC samples. Among CRC samples, *KRAS* was mutated in 2354 (48.1%). Most frequent codon 12 mutations were G12D in 731 samples (14.9%) and G12V in 522 (10.7%), followed by G12C in 167 (3.4%). *KRAS* mutations were more frequent in females than males (*p* = 0.003), however this difference was exclusive of non-G12C mutants (*p* < 0.001). *KRAS* mutation frequency was lower in the South and North regions (*p* = 0.003), but again *KRAS* G12C did not differ significantly (*p* = 0.80). In NSCLC, *KRAS* mutations were found in 1004 samples (21.4%). As opposed to CRC samples, G12C was the most common mutation in *KRAS*, in 346 cases (7.4%). The frequency of *KRAS* G12C was higher in the South and Southeast regions (*p* = 0.012), and lower in patients younger than 50 years (*p* < 0.001). *KRAS* G12C mutations were largely mutually exclusive with other driver mutations; only 11 NSCLC (3.2%) and 1 CRC (0.6%) cases had relevant co-mutations.

**Conclusions:**

*KRAS* G12C presents in frequencies higher than several other driver mutations, and may represent a large volume of patients in absolute numbers. *KRAS* testing should be considered in all CRC and NSCLC patients, independently of clinical or demographic characteristics.

**Supplementary Information:**

The online version contains supplementary material available at 10.1186/s12885-021-07884-8.

## Introduction

The field of precision oncology has revolutionized the way cancer is approached in the past two decades [1–3]. Throughout the years, numerous compounds have been developed targeting proteins responsible for the development and maintenance of cancer cells, with relevant implications in the clinic. In parallel, a better understanding of cancer biology and genetics enabled researchers to define predictive biomarkers that help oncologists select the most suitable targeted therapies for patients [4].

*RAS* genes were identified as the first oncogenes in 1982; specifically a single missense mutation in *HRAS* codon 12 was found in a bladder carcinoma cell line [5, 6]. Subsequently, the somatic origin of *RAS* mutations was confirmed, three isoforms were described and hotspots were found at codons 12, 13, and 61. *KRAS* is the most frequently mutated oncogene in cancers, with mutation rates of up to 96% in pancreatic cancers [7, 8], 54% in colorectal cancers (CRC) [9, 10], and 39% in lung adenocarcinomas [11]. RAS proteins belong to a superfamily of small GTPases that regulate fundamental intracellular signaling pathways involved in cell growth and survival [12, 13]. RAS missense mutations stabilize the protein in its active, GTP-bound state, which leads to sustained transduction of pathways including the MAPK [14–16].

In CRC, it has been demonstrated that *RAS* mutations are associated with lack of response to monoclonal antibodies against the epidermal growth factor receptor (EGFR), an upstream target related to RAS [17]. *RAS* testing is currently required in metastatic CRC in order to rule out the presence of missense mutations, and select patients for anti-EGFR antibodies [17]. In non-small cell lung cancer (NSCLC), *RAS* mutations have also been related to limited benefit from EGFR tyrosine kinase inhibitors [18]. Subsequently, *EGFR* mutations per se were shown to determine sensitivity to these inhibitors, and are mostly mutually exclusive with *RAS* [2]. Several other genomic alterations have been shown to determine sensitivity to specific targeted therapies in NSCLC [19]. Routine testing of gene mutations or fusions involving *EGFR*, *ALK*, *RET*, *ROS1*, *MET*, *BRAF*, *ERBB2*, and *NTRK* are recommended to select patients for targeted therapy [20].

KRAS G12C mutant has been exploited as a potential target for novel therapies. Indeed, the codon 12 cysteine residue may serve as a binding pocket for covalent inhibitors, stabilizing KRAS in an inactive, GDP-bound form [21]. Two novel compounds have been developed to act against KRAS G12C and are under clinical development, sotorasib (AMG 510) [21] and adagrasib (MRTX849) [22]. Understanding the frequency of *KRAS* G12C and co-occurring mutations is crucial to define strategies for cancer control. Herein we present a large review of CRC and NSCLC tumor samples submitted for *KRAS* testing in a clinical certified laboratory.

## Materials and methods

### Study design

We retrospectively analyzed deidentified data from CRC and NSCLC tumor samples submitted to *KRAS* testing in a clinical certified laboratory (Progenética, Grupo Pardini – Brazil) between 2017 and 2019. Available clinical data were reviewed and annotated. No patient had duplicated tumor samples analyzed. Samples were previously acquired for clinical purpose, included either biopsy or resection, and were all preserved in formalin-fixed paraffin-embedded (FFPE) tissue blocks. Following the laboratory routine, tumor slides were examined to confirm sample diagnosis and adequacy for sequencing. At least two 4–6 micra tumor slides were required, with tumor content of 5% or more. DNA was extracted using an automated protocol (QIAsymphony DSP DNA Mini Kit, Qiagen; Hilden, Germany), and quantified using the Qubit 2.0 fluorometer (Thermo Fisher Scientific, Waltham, Massachusetts, US). A minimum of 10 ng of genomic DNA was required for targeted sequencing.

CRC samples were tested for *KRAS* and *NRAS* mutations using pyrosequencing (Therascreen KRAS Pyro Kit and Therascreen RAS Extension Pyro Kit, Qiagen; Hilden, Germany), while NSCLC samples were tested for *KRAS*, *NRAS*, *BRAF*, and *EGFR* by next generation sequencing (NGS; Thermo Fisher Technology; Waltham, Massachusetts, United States). Co-occurring mutations were confirmed by an orthogonal method. The pyrosequencing was performed according to handbooks of Kits’ manufacturer (Therascreen KRAS Pyro® Handbook and Therascreen RAS Extension Pyro® V2 Kit Handbook). The following hotspot sites mutations were analyzed for *KRAS* and *NRAS*: codons 12–13, 61, 117 and 146. Reactions were run in the PyroMark Q24 instrument (Qiagen; Hilden, Germany) and analyzed using the software v2.0.7 in default mode. The NGS panel was customized to cover 17 amplicons of interest in hotspot regions at: *EGFR* exons 18 (codons 696–725), 19 (codons 729–761), 20 (codons 762–796 and 808–823) and 21 (codons 856–875); *KRAS* exons 2 (codons 6–37), 3 (codons 38–65) and 4 (codons 114–149); *NRAS* exons 2 (codons 4–30), 3 (codons 43–68) and 4 (codons 125–150); and *BRAF* exons 11 (codons 439–472) and 15 (582-610).

For NGS analysis, raw sequence data were mapped to the hg19 human reference genome using Torrent Mapping Alignment Program aligner. Torrent Coverage Analysis Plugin implemented in v5.0 of the Torrent Suite software (Thermo Fisher Scientific) was used to perform initial quality control and used to assess amplicon coverage for regions of interest. Regions were obtained after filtering of uniformity (> 80%), on-target reads (> 50%) and minimum mapped reads of 25.000. Variant calling was carried out using Ion Reporter v5.0 (Thermo Fisher Scientific) with the default setting of somatic parameters (minimum variant quality of 10, minimum coverage of 5×, maximum strand bias of 0.95, and minimum variant score of 6). The lowest limit of detection for low-frequency variants was 5%. Variants were annotated using the following databases: 5000Exomes V.1, Canonical RefSeq Transcripts v63, ClinVar v.1, COSMICv.67, dbSNP v.138, DGV v.20130723, DrugBank v.1, Gene Ontology, v. 1.218, OMIM v.03022014, Pfam v.26, PhyloP Scores v.1, RefSeq Functional Canonical Transcripts Scores v.4 RefSeq GeneModel v. 63. All identified variants were checked for correct nomenclature viewing the Integrative Genomics Viewer (IGV) alignment.

### Statistical analysis

Clinical and molecular data (age, gender, geographic region, histology, and mutation *status*) are described in absolute numbers and frequencies. Age is also provided as mean and standard error (SE). Categorical variables were compared using Pearson’s Chi-square test. *KRAS status* was classified as G12C mutant, non-G12C mutant (“KRAS Others”), and wild type. All comparisons included two-tailed tests, with level of significance set at 5%. All analyses were performed using IBM SPSS software (version 26.0; IBM Corporation, Armonk, NY, USA). All methods were carried out in accordance with relevant guidelines and regulations.

### Ethical considerations

The protocol was reviewed and approved by local Ethics Committee (Research Ethics Committee of the Minas Gerais Social Security Institute – IPSEMG). A waiver for the informed consent was also approved by the Research Ethics Committee of the Minas Gerais Social Security Institute since all patients had previously signed an authorization for testing, and data were collected retrospectively through molecular reports review. No information capable of identifying patients was used.

## Results

### Patient and sample characteristics

The dataset comprised 4897 CRC and 4686 NSCLC samples (Table 1). Mean age was 59.8 years (SE 0.186) and 65.6 years (SE 0.170) among CRC and NSCLC patients, respectively. Patient cases were well balanced according to gender in both cohorts (51.8 and 45.7% males, respectively). Brazilian Southeast and South regions were the most frequently represented, however all regions were present in the complete dataset. Adenocarcinoma was the most frequent histological subtype in both cases (87.8 and 81.4%, respectively). All *KRAS* G12C mutations were caused by a c.34G > T missense mutation.

**Table 1:** Patient and sample characteristics

Characteristics	CRC (*N* = 4897)	NSCLC (*N* = 4686)
Age (years), N (%)		
< 40	339 (6.9)	97 (2.1)
40–49	661 (13.5)	302 (6.5)
50–59	1241 (25.3)	922 (19.7)
60–69	1530 (31.2)	1572 (33.7)
> 70	1126 (23.0)	1776 (38.0)
Gender, N (%)		
Male	2535 (51.8)	2142 (45.7)
Female	2362 (48.2)	2544 (54.3)
Region, N (%)		
Southeast	3614 (73.8)	2599 (55.5)
South	641 (13.1)	672 (14.3)
Northeast	133 (2.7)	769 (16.4)
Middle West	407 (8.3)	449 (9.6)
North	102 (2.1)	197 (4.2)
Histology, N (%)		
Adenocarcinoma	4300 (87.8)	3814 (81.4)
Squamous cell carcinoma	–	19 (0.4)
NSCLC NOS	–	205 (4.4)
Unknown	597 (12.2)	648 (13.8)

Abbreviations: *CRC* colorectal cancer; *NSCLC* non-small cell lung cancer; *NOS* not otherwise specified. *Obs* seventeen NSCLC cases lacked information on age; hence only valid percentage is presented

### Tumor genotyping

**Fig. 1:**
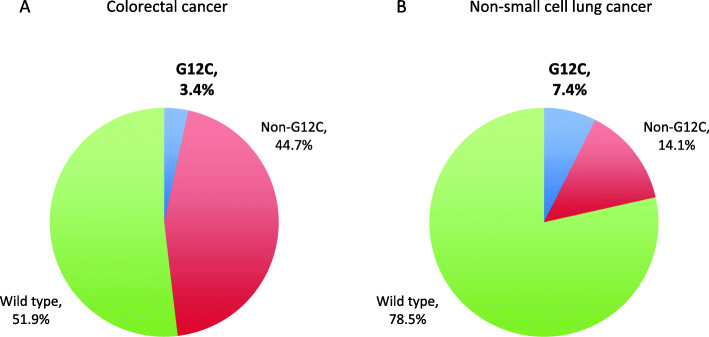
Frequency of KRAS G12C and non-G12C mutations in colorectal (**a**) and non-small cell lung cancer (**b**)

**Fig. 2:**
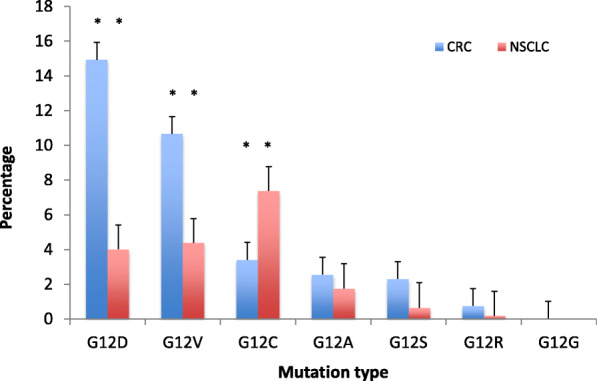
Distribution of KRAS codon 12 mutations in colorectal (CRC) and non-small cell lung cancer (NSCLC)

**Fig. 3:**
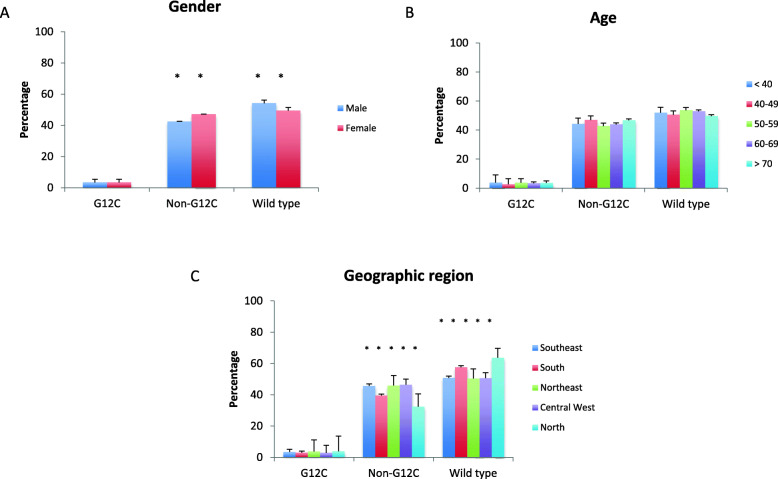
Frequency of KRAS G12C and non-G12C mutations according to gender (**a**), age (**b**), and geographic region (**c**) in colorectal cancer

Among 4897 CRC samples tested, *KRAS* was mutated in 2354 (48.1%). Most frequent hotspots were at codon 12 in 1696 samples (34.6%), codon 13 in 403 (8.2%), and codon 61 in 116 (2.4%). Most frequent codon 12 mutations were G12D in 731 samples (14.9%) and G12V in 522 (10.7%), followed by G12C in 167 samples (3.4%) (Figs. 1 and 2). Patients with *KRAS* G12C had a mean age of 60.2 years (SE 1.061), and were well distributed according to gender (51.9% males). The presence of *KRAS* mutations was significantly higher among females than males (50.5% vs 45.8%; *p* = 0.003), however this difference was exclusive of non-G12C mutants (*p* < 0.001). *KRAS* mutation frequency was significantly lower in the South and North regions (42.4 and 36.3%, respectively; *p* = 0.003), but again *KRAS* G12C did not differ significantly (*p* = 0.80). The frequency of *KRAS* G12C varied from 2.9% in the Middle West to 3.9% in the North. No association was seen between *KRAS status* and age (*p* = 0.481). The frequency of *KRAS* G12C varied from 2.6% for patients aged 40–49 years to 3.8% for patients younger than 40 (Fig. 3 and Supplemental Table 1).

**Fig. 4:**
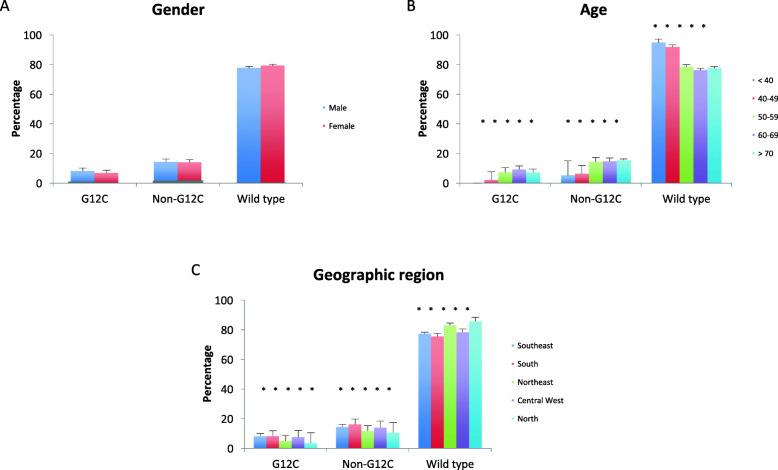
Frequency of KRAS G12C and non-G12C mutations according to gender (**a**), age (**b**), and geographic region (**c**) in non-small cell lung cancer

Among 4686 NSCLC samples, *KRAS* mutations were found in 1004 (21.4%), mostly at codon 12 in 875 (18.7%) and codon 13 in 64 (1.4%). The distribution of *KRAS* G12 mutations was significantly distinct from CRC data (*p* < 0.001; Figs. 1 and 2). As opposed to CRC samples, G12C was the most common mutation in *KRAS*, in 346 cases (7.4%), followed by G12V in 206 (4.4%) and G12D in 188 (4.0%). Mean age of *KRAS* G12C patients was 67.0 years (SE 0.486), and half were males (50.0%). In contrast to CRC data, gender was not associated with *KRAS* mutation *status* (*p* = 0.229). On the other hand, significant associations were described between *KRAS status*, region (*p* = 0.003), and age (*p* < 0.001). The frequency of *KRAS* G12C was higher in the South and Southeast regions (8.2 and 8.1%, respectively), as opposed to 5.1 and 3.6% in the Northeast and North, respectively (*p* = 0.012). *KRAS* G12C was less frequent at younger ages: 2.0% or lower in patients younger than 50 years, as opposed to 7.2% or higher in patients older than 50 (*p* < 0.001; Fig. 4 and Supplemental Table 2).

### Co-occurring mutations

Among NSCLC samples, *EGFR* mutations were detected in 1162 (24.8%), *BRAF* in 167 (3.6%) and *NRAS* in 27 (0.6%). Most frequent *EGFR* mutations were exon 19 deletions in 611 samples (13.0%), and exon 21 L858R point mutation in 326 (7.0%). *BRAF* mutations involved codon 600 in 86 samples (1.8%). *KRAS* G12C mutations were largely mutually exclusive with other driver mutations, however 21 cases (6.1%) presented reportable co-mutations (Table 2). *KRAS* G12C co-existed with *EGFR* mutations in 10 cases, *BRAF* in 4, and *NRAS* in 3. G12C co-occurred with other *KRAS* mutations in 5 cases. Nevertheless, 7 co-mutations were non-canonical, and 5 had an allelic frequency lower than 5%; hence only 11 (3.2%) NSCLC cases had clinically-relevant co-mutations. One sample presented with 3 mutated genes, including *KRAS* G12C (allelic frequency of 17%), *EGFR* S768I (18%), and *BRAF* V600E (5%).

**Table 2:** Synchronic mutations co-occurring with *KRAS* G12C among NSCLC samples

Genes	Amino acid change	Allelic frequency %)
*EGFR*	Exon 19 deletion (2 cases)	19 and 2.5
	L858R (2 cases)	4 and 2
	S768I^a^	18
	G719N	14
	T725M^b^/A597P^b^/R831H^b^/V774M^b^	49/13/3/7
*KRAS*	G13D (2 cases)	49 and 36
	G12S	17
	G12V	4
	Q22^b^	16
*BRAF*	V600E^a^ (2 cases)	7 and 5
	V600K	20
	G464E^b^	15
*NRAS*	Q61K	24
	G13V	14
	E132K^b^	NR

^a^ 1 case of co-existing *KRAS* G12C, *EGFR* S768I, and *BRAF* V600E; ^b^ non-canonical mutations. Abbreviation: *NR* not reported

In CRC, 3 samples presented with co-existing mutations in *KRAS*: G12S and G12V (2 cases); G12V and G12C (1 case). *NRAS* status was available for 2261 patients, of which 187 (8.3%) were mutated. The most frequent *NRAS* mutations involved codons 61 in 94 samples (4.2%), codon 12 in 76 (3.4%) and codon 13 in 14 (0.6%). There was no overlap between *KRAS* and *NRAS* mutations among CRC samples.

## Discussion

KRAS G12C has become a promising target for novel directed therapies in solid tumors [23], adding up a new role for routine *KRAS* testing. Given the lack of comprehensive and up to date information on the frequency of *KRAS* G12C and its characteristics of presentation, our group reviewed data from a large cohort of CRC and NSCLC samples (almost 10 thousand samples overall) submitted to *KRAS* testing in a clinical certified laboratory. Our data provide relevant input to guide future efforts in the field.

We described a frequency of *KRAS* G12C of 3.4% in CRC and 7.4% in NSCLC, which is higher than rates of several other driver mutations currently in clinical use to guide targeted therapies [24]. In absolute numbers, these figures represent a large volume of patients that may benefit from novel directed therapies. The percentages described herein are in line with COSMIC database, where a frequency of 2.6 and 6.9% were reported in colon and lung adenocarcinomas, respectively [16, 25]. In Brazil, a comprehensive review of 8234 metastatic CRC patients found *KRAS* mutations in 31.9% [26]. G12C was present in 7.9%, but ranged from 7.1% in the Southeast to 12.2% in the North region [26]. The frequency of *KRAS* G12C was slightly higher in females than in males (8.6 and 7.1%, respectively) [26]. No association was observed between age, gender, region, and KRAS G12C status in the current CRC cohort.

*KRAS* mutational data were described in a review of 513 lung adenocarcinomas from Brazilian patients profiled by NGS [27]. *KRAS* G12V and G12C were the most common mutations, in 6.9 and 6.7%, respectively. Analysis of clinical and demographic characteristics was not provided. In a review of 5738 NSCLC samples from Latin America, *KRAS* mutations were found in 14% of samples, however no details were offered on *KRAS* G12C [28]. The current NSCLC data adds up by providing detailed *KRAS* analysis, and comparisons to clinical and demographic information. Our group found that *KRAS* G12C was significantly more frequent in older patients, as well as those from South and Southeast regions. A plausible explanation is the higher proportion of tobacco-related diseases at older age, hence linking this risk factor to *KRAS* G12C-related NSCLC. Also, South and Southeast are the regions with highest consumption of tobacco in the country [29], reinforcing the relationship between tobacco and *KRAS* G12C-mutant NSCLC. South and Southeast are also regions with the highest income in the country; molecular assessment may be more appropriate in these areas.

In agreement with COSMIC [25], *KRAS* G12C was the most frequent mutation in NSCLC, while *KRAS* G12D and G12V were the most common in CRC. The clinical observation that *KRAS* mutations differ in position and type of substitution according to cancer type is an intriguing, and yet unexplained phenomenon [16]. It has been suggested that distinct carcinogens may act differently to cause specific *KRAS* mutations and create selective pressures that will guide the process of tumor initiation in each tissue type [16]. For instance, *KRAS* G12C mutation is universally caused by a single nucleotide substitution at position 34, G > T. This transversion is commonly found in mutational signatures associated with tobacco-related carcinogens in lung cancer [30]. On the other hand, *KRAS* G12D is caused by a G > A transition characteristic of CRC mutational signatures [30].

*KRAS* mutations are classic driver oncogenes, and rarely co-occur with other targetable mutations. In our dataset, 21 *KRAS* G12C-mutant NSCLC cases (6.1%) presented reportable co-mutations, however only 11 (3.2%) were canonical nucleotide variations with an allelic frequency of 5% or more. In a large database including 1078 *KRAS*-mutant NSCLC samples [31], *EGFR* mutations were found in 1.2%, *BRAF* in 1.4%, and *NRAS* in 0.5%. *KRAS* G12C was associated with a higher frequency of *ERBB2* amplification and *ERBB4* mutations, while *PTEN* and *BRAF* mutations were less common than in the total cohort [31]. The finding that 2 or more gene mutations may co-occur within the same tumor likely reflects a multiclonal presentation that leads to tumor heterogeneity [31]. *KRAS* mutations may also emerge as a mechanism of secondary resistance to targeted therapies [32]. *KRAS* mutations commonly present alongside mutations in *TP53*, *STK11*, and *KEAP1* and these co-mutations define distinct signature with potential clinical implications [33]. These genes were not assessed in our database.

The current study was based on laboratory reports; therefore limited clinical data were available. For instance, information on smoking status, disease stage, treatment, and outcomes were not provided. On the other hand, a large sample size was reached in a short and contemporary timeframe, which supports the reproducibility of our dataset. In addition, data analyzed was representative of the 5 geographic regions in the country. Brazil spans a large territorial extension and there may exist some degree of genetic background heterogeneity in the population. Differences in risk factors and disease presentation are expected, further supporting the strength of our results. It should be noted that North and Northeast regions were underrepresented in the current study, which may have caused an imbalance in the analysis. Future studies should focus on these regions to increase the power to confirm our findings. Lastly, modern sequencing platforms were applied in the current analysis, including pyrosequencing and NGS. The greater accuracy of these methods makes results more reliable than past publications in the field.

## Conclusion

The current dataset provides a comprehensive and contemporary overview of the molecular profile of *KRAS* G12C in both CRC and NSCLC. *KRAS* G12C presents in frequencies higher than several other driver mutations, and may represent a large volume of patients in absolute numbers. *KRAS* testing should be considered in all CRC and NSCLC patients, independently of clinical or demographic characteristics, in order to improve enrolment in clinical trials.

## Supplementary Information

**Additional file 1: Table S1** Distribution of colorectal cancer patients’ characteristics according to KRAS status. **Table S2** Distribution of non-small cell lung cancer patients’ characteristics according to KRAS status.

## Data Availability

Only limited and well described gene regions (hotspots) were sequenced for clinical purposes. Moreover, sequencing data generated were not meant for discoveries and no novel mutations were described. Therefore, sequencing data were not made available in a public database. The authors datasets analyzed during the current study are available from the corresponding author on reasonable request.
