# Supplementary material for: Molecular profile of KRAS G12C-mutant colorectal and non-small-cell lung cancer
Source: BMC Cancer. 2021 Feb 25;21:193. doi: 10.1186/s12885-021-07884-8 (PMC7905642; doi:10.1186/s12885-021-07884-8)
Supplement: Supplementary file 1 — Additional file 1: Table S1 Distribution of colorectal cancer patients’ characteristics according to KRAS status. Table S2 Distribution of non-small cell lung cancer patients’ characteristics according to KRAS status. [file 12885_2021_7884_MOESM1_ESM.pdf]

Molecular profile of *KRAS* G12C-mutant colorectal and non-small-cell lung cancer

Supplemental Table 1 - Distribution of colorectal cancer patients' characteristics according to *KRAS* status.

**Characteristics**	***KRAS* G12C**	***KRAS* Others**	***KRAS* WT**	**p-value**
Age (years)				
< 40	13 (3.8)	150 (44.2)	176 (51.9)	0.727
40-49	17 (2.6)	310 (46.9)	334 (50.5)	0.464
50-59	46 (3.7)	530 (42.7)	665 (53.6)	0.142
60-69	50 (3.3)	671 (43.9)	809 (52.9)	0.252
> 70	41 (3.6)	526 (46.7)	559 (49.6)	Ref
Gender, N (%)				
Male	86 (3.4)	1,074 (42.4)	1,375 (54.2)	Ref
Female	81 (3.4)	1,113 (47.1)	1,168 (49.4)	**0.003**
Region, N (%)				
Southeast	127 (3.5)	1,651 (45.7)	1,836 (50.8)	Ref
South	19 (3.0)	253 (39.5)	369 (57.6)	**0.007**
Northeast	5 (3.8)	61 (45.9)	67 (50.4)	0.986
Middle West	12 (2.9)	189 (46.4)	206 (50.6)	0.824
North	4 (3.9)	33 (32.4)	65 (63.7)	**0.028**

Abbreviations: WT, wild type; Ref, reference.

Supplemental Table 2 - Distribution of non-small cell lung cancer patients' characteristics according to *KRAS* status.

**Characteristics**	***KRAS* G12C**	***KRAS* Others**	***KRAS* WT**	**p-value**
Age (years)				
< 40	0 (0.0)	5 (5.2)	92 (94.8)	**<0.001**
40-49	6 (2.0)	19 (6.3)	277 (91.7)	**<0.001**
50-59	67 (7.3)	132 (14.3)	723 (78.4)	0.788
60-69	144 (9.2)	230 (14.6)	1,198 (76.2)	0.114
> 70	128 (7.2)	272 (15.3)	1,376 (77.5)	Ref
Gender, N (%)				
Male	172 (8.1)	304 (14.2)	1,665 (77.7)	Ref
Female	173 (6.8)	355 (14.0)	2,016 (79.2)	0.229
Region, N (%)				
Southeast	211 (8.1)	375 (14.4)	2,013 (77.5)	Ref
South	55 (8.2)	109 (16.2)	508 (75.6)	0.497
Northeast	39 (5.1)	91 (11.8)	639 (83.1)	**0.002**
Middle West	34 (7.6)	63 (14.0)	352 (78.4)	0.892
North	7 (3.6)	21 (10.6)	169 (85.8)	**0.015**

Abbreviations: WT, wild type; Ref, reference.
